# “Hesitating and Puzzling”: The Experiences and Decision Process of Acute Ischemic Stroke Patients with Prehospital Delay after the Onset of Symptoms

**DOI:** 10.3390/healthcare9081061

**Published:** 2021-08-19

**Authors:** Pao-Yu Wang, Lee-Ing Tsao, Yu-Wei Chen, Ying-Tao Lo, Hui-Lin Sun

**Affiliations:** 1 Department of Nursing, MacKay Junior College of Medicine, Nursing and Management, New Taipei City 25245, Taiwan; s562@mail.mkc.edu.tw; 2 Department of Nursing, National Taipei University of Nursing and Health Sciences, Taipei 112303, Taiwan; leeing.tsao@gmail.com; 3 Department of Neurology, Neuroscience Center, Landseed International Hospital, Taoyuan 324609, Taiwan; chenyw@landseed.com.tw; 4 Department of Neurology, National Taiwan University Hospital, Taipei 100225, Taiwan; 5 Department of Division of Marketing Planning, Landseed International Hospital, Taoyuan 324609, Taiwan; locherry@landseed.com.tw

**Keywords:** grounded theory, prehospital delay, acute ischemic stroke, rt-PA

## Abstract

Despite campaigns to increase public awareness of stroke symptoms by advocating FAST (Face-Arms-Speech-Time), some stroke patients still show delays in the recognition of and response to stroke symptoms and miss the golden first 4.5 h to receive rt-PA (recombinant tissue plasminogen activator) treatment. The aim of this study was to explore how acute ischemic stroke patients with prehospital delay seek help and undergo the decision process before arriving at the hospital. A qualitative approach using a grounded theory was applied. There were 24 ischemic stroke patients recruited by purposive sampling. Our main findings were: “Hesitating and puzzling” was the core category to describe and guide the process of acute ischemic stroke patients with prehospital delay. During the process, “Awareness the sudden change of physical sensation and/or function” was the antecedent category. In the prehospital delay experience, the following five interaction categories were identified: (1) “Self-judgment and interpretation according to previous experience,” (2) “Puzzling and doubting—it may only be a minor problem,” (3) “Self-treatment or seeking medical attention nearby,” (4) “Unexpected symptoms getting worse” needing immediate advanced medical help and (5) “Rushing to ER with different transportation—self-alerting that serious disease is coming.” Eventually, the patients “Regret to delay seeking treatment and become a disable person.” The process of prehospital delay provides some hidden cues for patients to increase their knowledge about strokes. The study emphasizes the importance of educating community residents about identifying stroke symptoms, breaking the myth of folk therapy, and seeking medical attention immediately. These results will assist healthcare providers by offering references for designing patient-centric educational strategies for preventing stroke prehospital delay to improve the quality of stroke medical care.

## 1. Introduction

Prehospital delay is defined as the prolonged time from symptom onset to arrival at a hospital; it can result in the failure to receive rt-PA (recombinant tissue plasminogen activator) on time for patients with ischemic cerebrovascular accidents [1,2]. In the UK, the Department of Health has informed that stroke patients should be treated on time and be assessed using “Stroke-Act FAST (Face-Arms-Speech-Time)” [3,4]. Although progress has been made in stroke treatment, the study indicated that only a few patients have access to rt-PA, resulting in thrombolysis rates of 1.3% to 8.4% [5,6]. One study suggested that the public policy aim of the utilization rate of intravenous thrombolysis should be more than 12% to have a significant population effect on disability [7]. Many studies have reported on the effectiveness of rt-PA treatment and global guidelines recommending the use of rt-PA in selected patients [8,9]. However, only a small proportion of patients (1.05% to 8.6%) receive thrombolysis treatment [10,11,12,13].

Some stroke patients have different symptoms and severity inconsistent with the FAST-promoted symptoms, affecting the patient’s decision to seek medical treatment and leading to extended medical treatment [14,15]. A further explanation for the limited perceived impact might be the description of the displayed symptoms using the FAST mnemonic [14]. Prehospital delay is an important cause of the inability of patients with ischemic stroke to obtain thrombolytic therapy at the appropriate time [16]. It has also become an important challenge in the management of stroke care [9]. Delays in medical treatment can be caused by a lack of recognition of stroke symptoms, failure to immediately treat when symptoms occur, transfer from general clinics to hospitals, and failure to use EMS (emergency medical services) [2,17,18]. More than two-thirds of patients failed to use EMS, consequently delaying their arrival at the hospital [18]. However, previous studies have demonstrated that the reasons for prehospital delays for stroke patients were a lack of initial recognition of stroke symptoms, unavailable immediate treatment, and not obtaining emergency stroke medical services [2,16,17,18].

To date, studies exploring the experiences and needs of acute ischemic stroke patients with prehospital delay after the onset of symptoms have not emphasized how time and/or ethnocultural background affect the ability of Taiwan stroke survivors to obtain the information, support, and services that they need to reintegrate into the community in the Asia context. Moreover, prehospital delay is an individual and complex phenomenon, and each stroke patient may show differences in experience seeking prehospital help. Therefore, the construction of a descriptive theory of subjective experiences is crucial for the care of acute ischemic stroke patients with prehospital delay after the onset of symptoms; a substantive theory grounded in experiences during the personal transition period under specific circumstances provides considerable potential to allow us to understand more comprehensively. Hence, classic grounded theory [19,20] was used to reveal the transition process. The aim of this study was to establish a descriptive theory of seeking help and undergoing the decision process from the subjective experience of acute ischemic stroke patients with prehospital delay in Taiwan. With the ultimate goal of helping to enhance the clinical/community care quality in future cases requiring appropriate healthcare.

## 2. Materials and Methods

### 2.1. Study Design

We conducted a grounded theory study of perceptions and processes of seeking help and decision-making before arriving at a hospital among acute ischemic stroke patients who did not receive rt-PA therapy due to prehospital delay. “Grounded theory” was commonly used when discovering a new perspective on individuals experiencing the phenomenon under study [21]. Therefore, it is appropriate to apply this method to explore and explain the process and experiences of prehospital delay among acute ischemic stroke patients in Taiwan.

### 2.2. Participants and Setting

Purpose sampling techniques were used in this study. In grounded theory, the sample size depends on the saturation of the analysis of the theme and whether there were repeated ideas or materials in discussion. If it does not lead to a new theme, it indicates saturation. The following criteria were used to select research participants: (a) at least 20 years, (b) diagnosis of the first stroke by a neurologist, (c) prehospital delay of more than 4.5 h before ischemic stroke patients with acute symptoms arrived at the emergency department with clear consciousness and normal language understandability, and (d) patients who could communicate in Taiwanese or Mandarin or Hakka and who agreed to be interviewed.

### 2.3. Data Collection

The recruitment period was from May 2018 to January 2019. The researchers visited the neurology clinics of regional hospitals to screen 26 patients who had met the inclusion and exclusion criteria of this study. Two patients refused to attend because they had no time to interview and were rushed to rehabilitation, and 24 participants agreed to participate in the interview. The researchers took the participants to an independent outpatient room for interviews to ensure the privacy of participants and facilitate the exploration of their experiences and perspectives. The interview process was anonymously recorded. The interviews averaged 20–40 min in length. The topic guides were developed by the study team according to the research purpose and reviewing the literature [22]. The topic guides content included the following questions: Could you tell me what happened to you when you were brought to the hospital? What were the symptoms? Why did you think you had these symptoms? What do you do to deal with these symptoms? What did the people around you do to help you? Under what circumstances did you decide to seek medical treatment? Could you tell me about the process of seeking medical treatment? How did you feel when you were taken to the hospital? What do you do when your symptoms don’t improve? What do you think is the lack of knowledge about prehospital treatment for stroke? Do you have any other experience you would like to share? At the end of each interview, the researcher listened to the entire interview recording, transcribed the interview process, and found meaningful ideas immediately. To ensure the accuracy of the transcription, the research team compared the transcription of the interview with the recording.

### 2.4. Data Analysis

In grounded theory, there are several steps for data analysis, including open coding, axial coding, and selective coding using the constant comparative method to analyze the interview content, and the theory is generated from the data analysis [19,20,23]. First, the content after the interview was written verbatim, from which descriptive sentences were formed that directly reflected respondents’ prehospital delay behavior. Each meaningful sentence was coded and compared with each other and then assigned to a suitable category according to attribute and orientation. Sub-categories were also assigned under each category. Next, axial coding was performed according to attribute and orientation linking category and sub-category to determine the relationship between each category. The categories and sub-categories were linked by continuous comparison to allow different categories to be related to each other. Finally, selective coding was performed to create a complete storyline of the whole research phenomenon under the guidance of the core category and explain the relationship among the antecedent categories, interaction categories, and outcome categories within a theoretical framework [20,21]. Data were collected until the point in coding when we found that no new codes occurred in the data, indicating that the data were saturated [24]. In total, 54 significant statements were produced by the 24 patients. The data were analyzed immediately after each interview and collected until all the genera were saturated. In other words, no new category was generated in the current interview content. In addition, the modified Rankin scale (mRS) was used to measure the degree of disability in patients who have had a stroke [25]. The researcher recorded the mRS score from patients’ medical charts before the interview.

### 2.5. Rigor

Research rigor is defined as the trustworthiness of a qualitative study. Four criteria are required to enhance trustworthiness: credibility, transferability, dependability, and confirmability [26]. In terms of credibility, credibility was confirmed by utilizing open-ended questions in interviews and by verifying whether the researcher heard the participants’ responses correctly. In addition, validation was obtained from participants who were keen to participate and share their in-depth subjective experience in this study. The process was implemented by asking three stroke patients to evaluate the overall results in order to examine the credibility of the results. The completed verbatim transcript of the interview was confirmed by the stroke patient to maintain authenticity; 24 stroke patients expressed the same experience with verbatim content according to their description. Regarding transferability, the participants recruited in this study were from various backgrounds to enable the researcher to obtain sufficient information from the participants to apply these findings to acute ischemic stroke patients. In terms of dependability, the researcher and senior nursing expert were involved in the data collection and analysis, and they constantly compared and recorded the transcripts to establish dependability. Finally, the researcher discussed and confirmed the coding and categorization according to the interview records to ensure the confirmability of the study.

### 2.6. Ethical Consideration

The Research Ethics Committee of the Landseed International Hospital Institutional Review Board (18-010-B1) approved this study. Before the interview, the researcher introduced himself to the participants, explained the topic and purpose of the interview, and reviewed the interview methods (including the recording of the process), possible problems, expected interview time, and benefits of the research. Participation was voluntary, and interviews were not conducted until consent was obtained.

## 3. Results

**Figure 1:**
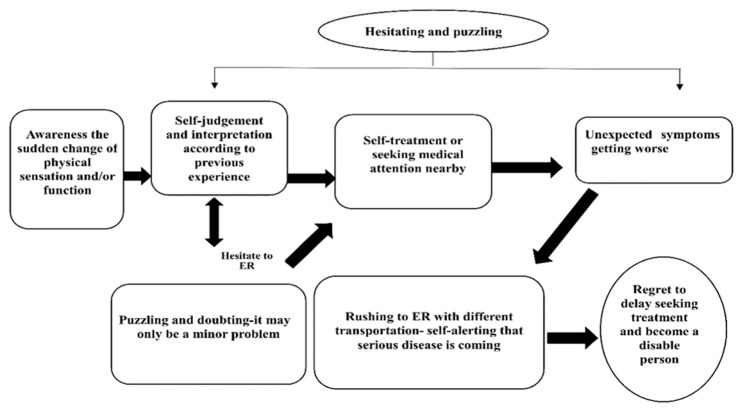
The theoretical framework.

Twenty-four first-time ischemic stroke patients participated in comprehensive interviews: 16 men and 8 women. The average age was 60.8 years. The mean duration from the onset of symptoms to arrival at the hospital was 34 h. The modified Rankin scale ranged from 1 to 3 points (indicating mild to moderate dysfunction, such as hemiparalysis and speech disturbance). The demographic data are shown in Table 1. This research was based on grounded theory. Figure 1 shows the theoretical framework. Among the analysis, “Hesitating and puzzling” was the core category to describe and guide the process of acute ischemic stroke patients with prehospital delay. During this process, “Awareness the sudden change of physical sensation and/or function” was the antecedent category to trigger the experience in the selection of treatment for the symptoms of patients with acute stroke. In the prehospital delay experience, the following five interaction categories were identified: (1) “Self-judgment and interpretation according to previous experience,” (2) “Puzzling and doubting—it may only be a minor problem,” (3)“Self-treatment or seeking medical attention nearby,” (4)“Unexpected symptoms getting worse,” and (5) “Rushing to ER with different transportation—self-alerting that serious disease is coming.” When patients with acute stroke went through this process, they all believed the problem may be minor and took the approach they were familiar with. When the symptoms worsened and a stroke seemed possible, they went to the emergency room immediately. The whole experience of medical treatment occurred in the context of “Hesitating and puzzling.” Eventually, they were “Regret to delay seeking treatment and become a disable person.” Quotations are identified by participant number from 1 to 24 (e.g., P1) (the “P” means “patient”).

### 3.1. Hesitating and Puzzling

“Hesitating and puzzling” means that the experiences of seeking help and decision-making before arriving at the hospital among the stroke patients who missed receiving rt-PA therapy were due to prehospital delay. Acute ischemic stroke patients in the process were in a state of hesitating and puzzling due to the inability to recognize stroke symptoms. Then, through reasonable self-judgment, these patients addressed issues in a familiar way, but the symptoms did not improve, leading to the results of prehospital delay, as described in the case:

*When I went shopping on foot, I kicked my left foot all the way to the floor because it felt like it was weak and numb. I thought it was just too tired. Because my hands and feet still moved, I didn’t think it was a stroke. So my husband took me to get a massage … Stick the Chinese medicine patches on the thigh, and I went home to sleep after folk therapy. I thought it should be all right. I didn’t expect to wake up the next morning and find that my left foot couldn’t be lifted. I was scared … I called my son to my room right away … When my son saw me like this, he got me up immediately. I didn’t change my clothes. I limped to his car. He drove me to the hospital very fast … When I got to the hospital, the doctor said it was a stroke … but we missed the golden cure. I was very depressed … because there was no chance of a thrombolytic injection (P15)*.

### 3.2. Awareness the Sudden Change of Physical Sensation and/or Function

“Awareness the sudden change of physical sensation and/or function” means that the patient suddenly felt limb numbness, drooling, dizziness, headache, or limb weakness, facial asymmetry, as described in the case:

*At six o’clock in the morning, when I got up, I couldn’t stand stably. I felt that my left foot had no strength to walk, so I leaned to one side. The glass bottle in my left hand fell to the ground … My mouth was dripping all the time (P1)*.

*When I walked, I suddenly felt dizzy and unstable walking. Both hands and feet had no strength, and my hands and feet on one side were numb (P2)*.

*When I was talking to my wife, my face suddenly turned crooked (P4)*.

### 3.3. Puzzling and Doubting—It May Only Be a Minor Problem

“Puzzling and doubting—it may only be a minor problem” means that the patients thought these symptoms were just a minor physical problem because the patients believed that there was no risk factor for stroke (e.g., they were young, not fat, not high triglyceride levels), had no symptoms of stroke (e.g., could still move their limbs), or took medicine at ordinary times to prevent stroke, as described in the case:

*If I had a stroke, it seemed that I could not move my hands and feet, that’s it … I could still move! (P7)*.

*I didn’t have a problem with high triglyceride levels. It’s just high blood sugar … It’s just the numbness of the feet … I’m not fat either (P12)*.

*I want to say that I am so young … maybe it’s work that causes numbness and weakness (P14)*.

*I take medicine to prevent stroke every day … It’s impossible to have a stroke (P23)*.

### 3.4. Self-Judgment and Interpretation According to Previous Experience

“Self-judgment and interpretation according to previous experience” means that the patient thought that the physical changes were due to past illness experiences, e.g., cold, fasciitis, hand cramps, nerve compression, inner ear disorders, or daily life experience, e.g., hot weather or being too tired, as described in the case:

*Because of the problem of nerve compression, I may be a little slow in walking (P5)*.

*This foot had fasciitis before … I wonder if it caused it (P10)*.

*Dizziness …I thought it was an inner ear disorder (P13)*.

*It’s too hot to be comfortable … Maybe it’s too tired to drive (P11)*.

### 3.5. Self-Treatment or Seeking Medical Attention Nearby

“Self-treatment or seeking medical attention nearby” means that patients took rest, used folk therapy (e.g., massage, Chinese medicine patches, bloodletting with scissors or needles), or went to a nearby pharmacy or clinic for help. The culture of folk therapy has had an extremely strong influence on some patients, as described in the case:

*Massage thighs or legs … used scissors to stab the index finger and middle finger, pressing philtrum (P10)*.

*I kicked my left foot all the way to the floor … it was weak and numb … my husband took me to get a massage …stick the Chinese medicine patches on the thigh (P15)*.

*Went to the nearby pharmacy to take Chinese medicine … went to the clinic for a sore injection (P17)*.

### 3.6. Unexpected Symptoms Getting Worse

“Unexpected symptoms getting worse” means that when patients with dizziness did not improve and showed vomiting, fell, one-sided hands that were unable to hold things, feet were unable to lift, more numbness in the hands and feet, or obvious speech disturbance and facial asymmetry, as described in the case:

*I still felt dizzy and start to vomit. Speaking was very unclear … The wife said that my face was asymmetric (P2)*.

*When I went to the toilet, I suddenly fell to the ground when my feet were weak … One foot couldn’t walk. It’s walking by dragging (P17)*.

*Hands and feet became more numb, holding things in the hands couldn’t hold, fell on the ground … couldn’t lift the left foot when walking (P22)*.

### 3.7. Rushing to ER with Different Transportation—Self-Alerting That Serious Disease Is Coming

“Rushing to ER with different transportation—self-alerting that serious disease is coming” means that when patients found that they might have had a stroke or became seriously ill, they drove, called a taxi to the hospital through their family/friends, rode their own motorcycle/drove to the hospital, or called 119 to call an ambulance to the hospital, as described in the case:

*Told my wife to drive over to the hospital immediately … It must be a big problem (P7)*.

*Stroke … My son’s wife quickly called a taxi to take me to the hospital (P17)*.

*The doctor said that there might be a stroke… so I drove to the emergency room (P14)*.

*It’s a stroke. My wife was looking for help from the neighbor, who carried me from the second floor to the first floor, my wife called 119 and called an ambulance to the emergency room (P12)*.

### 3.8. Regret to Delay Seeking Treatment and Become a Disable Person

“Regret to delay seeking treatment and become a disable person” means that the patient felt sad and melancholic when facing physical disability and regretted failing to arrive at the hospital in time to miss the golden emergency treatment time, as described in the case:

*I have a stroke now, like this … (with tears), my hands and feet are weak and I need my husband’s help now (P9)*.

*I didn’t dare to go out when I just came home from hospital. I stayed in my room … didn’t want to talk to anyone … I didn’t think it was a stroke at the time, so I didn’t come to the hospital right away … It’s a pity that I’ve delayed my golden therapy, because it’s going to take a lot of time to recover now, and I would cry when I thought of becoming like this (P10)*.

*I really regret coming to the hospital so slowly … My daily life now depends on the help of my family … If I come early, maybe I could get a thrombus injection (P17)*.

## 4. Discussion

This study illuminates the experiences of seeking help and decision-making before arriving at the hospital among acute ischemic stroke patients who missed receiving rt-PA therapy due to prehospital delay. The stroke patients with prehospital delay were full of hesitation and were puzzled before arriving at the hospital. The findings were similar to those of a previous study in which the main cause of delay in reaching the hospital was identified as indecision [27]. In the current study, the patient’s self-awareness of stroke symptoms in the head, face, or limbs reminds us that we should strengthen self-discovery, the cognition of early symptoms of stroke, and timely arrival at available and appropriate hospitals. It is better for local clinic staff to strengthen their understanding of on-the-job education to reduce unnecessary prehospital delays.

These patients with delay in medical treatment showed signs of the sudden change of physical sensation and/or function similar to other studies [28,29], indicating that the initial symptoms of stroke patients who are delayed in seeking medical care include weakness on one side, headache, slurred speech, blurred vision, and wry mouth. When a stroke suddenly occurred and the patient could continue to perform normal daily activities, the initial neurological symptoms were ignored (e.g., one side of the limb is weak, slurred speech, blurred vision, diplopia, or headache), the patient chose not to seek medical treatment immediately [28]. Therefore, the promotion of the Face-Arms-Speech-Time formula is not only needed in assessing stroke symptoms but also to increase public awareness of other neurological symptoms associated with stroke and to emphasize that each patient may have different neurological symptoms and severity of symptoms.

When neurological symptoms occurred, the patients would make a self-judgment and interpretation according to previous experience; they believed that there was no risk factor for stroke or that the limbs could still move, so the body likely only had a small problem. Mackintosh et al. [15] pointed out that stroke patients will try to match the symptoms with the diseases they know, often leading patients to misunderstand their stroke symptoms, such as they think that shoulders not being able to move are caused by nerve compression or that it is necessary to have a severe headache and then the failure of movement of a part of the body to develop a stroke. O’Connell and Hartigan [28] pointed out that patients tend to think the symptoms of stroke are caused by a migraine or dizziness when the patient has a headache. People’s perception of symptoms can be influenced by a previous experience of illnesses and the cultural norms and values of their communities [30]. Therefore, it is recommended that when advocating how to respond to stroke symptoms, it should be pointed out that stroke symptoms will occur suddenly and that the neurological symptoms of stroke patients are different from the physical and neurological symptoms caused by aging or other diseases.

The results of this study found that the patients would take a break or go to a nearby pharmacy to take medication, a muscle relaxant, or an analgesic injection to treat the physical symptoms, similar to what was found in other studies [28,29]. Thus, patients with delayed medical treatment would wait for a while after symptoms appear, during which time they would allow themselves to lie down, rest, sleep, or continue their normal life, as they expected the symptoms to disappear or considered their actions may contribute to symptom relief [28,29]. Ahasan et al. [27] pointed out that stroke patients choosing to first visit the general clinic or pharmacy to seek medical treatment will cause delays in treatment. When most people are unwell, they will go to a familiar and trustworthy family doctor because they believe they can obtain credible medical information and advice [15]. However, it is quite uncertain whether these nearby medical clinics have sufficient professional knowledge for strokes. Notably, this study also found that some stroke patients followed the advice of friends or advertisements to use scissors or acupuncture to bleed the hands and feet, press the philtrum, or use massage to deal with stroke symptoms. However, using folk therapy to deal with stroke symptoms has no similar findings in the literature of other Western countries [27,28,29]. This may be related to the regular use of traditional Chinese medicine (TCM) by Taiwanese adults [31,32,33]. Because some parts of TCM are similar to folk therapy, it is reasonable for TCM users to also use folk therapy [34]. However, folk therapy still cannot prove its definite medical effect [35]. Taiwan’s Health Promotion Administration, the MOHW (Ministry of Health and Welfare) [36], has indicated that if bloodletting is performed as stroke first aid, the blood pressure will fall to dangerously low levels, accelerating brain cell death.

However, when these patients’ unexpected symptoms become worse, most of them went to the hospital in the car of a family member or a friend or by taxi, or they rode a motorcycle or drove to the emergency room of the hospital. Similar to other studies, patients will seek immediate medical treatment when they have severe stroke symptoms [16,17]. It was pointed out by a research survey of Wongwiangjunt et al. [37] that 83.4% of patients with acute stroke took a family car or taxi to the hospital, and only 16.6% of patients used an ambulance. Previous studies have indicated that access to emergency medical services can shorten the time to delay in prehospital care for patients with stroke [2,17,18].

The results of this study showed that when the patients arrived at the emergency department, they felt regret for missing the golden time of emergency treatment, as they did not go to the hospital immediately, and subsequently felt sad, grieved, and depressed about facing physical disability. Harrison et al. [29] pointed out that stroke patients must realize that they have to arrive at the hospital within a limited time to obtain rt-PA medication, increasing their frustration with delays in the medical process. Thus, there is a significant correlation between stroke severity and post-stroke depression [38]. About one-third of stroke patients have symptoms of depression over time [39].

The delay in the treatment of acute ischemic stroke patients before they arrive at the hospital is mainly due to patients lacking the correct perception of recognition and treatment of stroke. This study provides deeper insight into the subjective experiences of acute ischemic stroke patients who missed receiving rt-PA treatment due to prehospital delays. Eliminating these delays would help health care providers more effectively by helping stroke patients to receive rt-PA in a timely manner. It is crucial to advocate more for prehospital self-judgment and self-management for acute ischemic stroke patients in addition to FAST.

The study area was limited to acute ischemic stroke patients who were delayed in seeking medical treatment in a regional hospital in northern Taiwan. Therefore, it is impossible to infer all stroke patients who have delayed medical treatment in Taiwan. The stroke patients who had aphasia and were too late to seek medical treatment were excluded from the study, so their experience was not presented. Despite the above limitations, our research still provides relevant factors leading to the delay in the treatment of patients with first-episode stroke. It is hoped that the findings of the study can serve as a reference for the government to develop strategies to improve the delay of stroke patients in obtaining medical treatment. Future research should further design intervention measures to improve the ability of the community residents to correctly manage strokes.

## 5. Conclusions

Acute ischemic stroke patients with prehospital delay were full of hesitation and were puzzled before arriving at the hospital. “Hesitation and puzzling” was the core category, and the causes of delay were the inability to recognize stroke symptoms, incorrect self-treatment, choosing to go to the clinic first, and failure to use emergency medical services. Our research emphasizes the importance of educating community residents about identifying stroke symptoms and correct stroke treatments, allowing the public to understand that stroke symptoms will have different neurological symptoms due to different parts of the brain affected by stroke. It was necessary to provide a common understanding of the related basilar artery and stroke neurological symptoms posterior to cerebral vascular circulation. Breaking the myth of folk therapy (e.g., bloodletting, massage, and so on) will be the key to helping people correctly identify stroke symptoms and quickly seek medical treatment. Therefore, to improve the delay of medical treatment for patients with stroke, in addition to actively advocating FAST to the community residents, it is imperative to provide education to identify the symptoms related to strokes and correctly manage strokes.

## Figures and Tables

**Table 1:** Characteristics of the participants.

Case No.	Gender/Age (Years)/Education Level	Diagnosis	History	MRS/*Time
1	Male/60/ Vocational high school	Right medial cerebellum infarcts	HTN; DM; hyperlipidemia	1/28 h
2	Female/62/ Vocational high school	Left pons infarcts	Arrhythmia	2/48 h
3	Male/63/ Junior college	Left pons infarcts	HTN; hyperlipidemia	2/4 h
4	Female/56/ Primary school	Right basal ganglia, right insular cortex, and right F-T-P lobes infarcts	Heart disease	2/9 h
5	Male/73/ Vocational high school	Right putamen and left centrum semiovale infarcts	HTN; hyperlipidemia	2/5 h
6	Male/64/ Vocational high school	Right corona radiata and right frontoparietal infarcts	DM	2/54 h
7	Male/52/ Graduate school	Cerebral infarction due to stenosis of right middle cerebral artery	HTN; hyperlipidemia	2/7 h
8	Male/67/ Primary school	Left internal capsule infarcts	HTN	2/72 h
9	Female/58/ Vocational high school	Right thalamic infarcts	DM; hyperlipidemia	2/26 h
10	Male/56/ Middle school	Left thalamus infarcts	Hyperlipidemia	2/22 h
11	Female/61/ Primary school	Right cerebral infarcts	HTN	2/28 h
12	Male/62/ Primary school	Left cerebellar infarcts	HTN Hyperlipidemia	3/48 h
13	Male/64/ Junior college	Right thalamus infarcts	Hyperlipidemia	2/72 h
14	Male/54/ Vocational high school	Left striatocapsule infarcts	Hyperlipidemia	1/72 h
15	Female/68/ primary school	Right pons, infarcts	HTN; hyperlipidemia	2/48 h
16	Male/63/ primary school	Left thalamus infarcts	Hyperlipidemia	1/24 h
17	Male/66/ primary school	Left pons infarcts	HTN; DM; hyperlipidemia	2/72 h
18	Male/65/ Middle school	Right posterior basal ganglia and corona radiate infarcts	DM	2/6 h
19	Male/63/ Vocational high school	Right periventricular white matter infarcts	HTN; DM; hyperlipidemia	2/5 h
20	Female/58/ Vocational high school	Left cerebral infarcts	HTN; DM; hyperlipidemia	3/48 h
21	Male/53/ Vocational high school	Right MCA infarcts	Hyperlipidemia	3/20 h
22	Female/58/ Vocational high school	Right MCA infarcts	HTN; hyperlipidemia	2/18 h
23	Female/57/ Middle school	Left pons infarcts	HTN; DM	2/72 h
24	Male/58/ Middle school	Right MCA infarction	HTN; hyperlipidemia	2/9 h

Note. MRS, Modified Ranking Scale; * Average time from symptom onset to hospital arrival; HTN, hypertension; MCA, middle cerebral artery; DM, diabetes mellitus.

## Data Availability

Not applicable.
